# The Vaginal Microbiome and Host Health: Implications for Cervical Cancer Progression

**DOI:** 10.3390/ijms27020640

**Published:** 2026-01-08

**Authors:** María del Carmen Lagunas-Cruz, Arturo Valle-Mendiola, Isabel Soto-Cruz

**Affiliations:** Laboratorio de Oncología Molecular, Unidad de Investigación en Diferenciación Celular y Cáncer, FES Zaragoza, Universidad Nacional Autónoma de México, Batalla 5 de Mayo s/n Col. Ejército de Oriente, Mexico City C.P. 09230, Mexico; lagunascruzmaryc@comunidad.unam.mx (M.d.C.L.-C.); arturo.valle@zaragoza.unam.mx (A.V.-M.)

**Keywords:** microbiome, dysbiosis, *Lactobacillus*, human papillomavirus, cervical cancer

## Abstract

The vaginal microbiome plays a crucial role in maintaining host health by preserving a balanced microenvironment. Nevertheless, the definition of a “normal” vaginal microbiome remains controversial, as its composition varies depending on factors such as ethnicity and geographical origin. In most cases, members of the genus *Lactobacillus* predominate in healthy vaginal microbiomes, protecting against potential pathogens through specific mechanisms such as the secretion of lactic acid and bacteriocins, among others. A reduction in *Lactobacillus* abundance, accompanied by an increase in anaerobic organisms, predisposes the host to the development of various pathologies. Among these pathologies is infection with human papillomavirus (HPV) and the subsequent development of cervical cancer. A progressive decline in *Lactobacillus* has been observed as the lesion advances in different populations worldwide. In the case of the Mexican population, several *Lactobacillus* have been reported in healthy microbiomes: *L. gasseri*, *L. fermentum*, *L. rhamnosus*, *L. jensenii*, *L. crispatus*, *L. delbrueckii*, *L. acidophilus*, and *L. brevis.* In contrast, genera reported in dysbiosis include *Sneathia*, while *Brevibacterium aureum* and *Brachybacterium conglomeratum* have been associated with HPV16 infection and/or SIL. The mere presence of some bacteria is not sufficient to modulate the cellular activity of host cells; therefore, the expression, production and activity of different proteins could be affected by the vaginal microbiome. The impact of the microbiome on host cell function is the result of different metabolites produced by the bacteria, which suppress or activate different signaling and metabolic pathways. The molecular interactions between the host and microbiome, as well as their role in cervical carcinogenesis, are still unknown. In this review, we focus on the vaginal microbiome, HPV, and the impact that the interaction of the microbiome with HPV has in cervical cancer development.

## 1. Introduction

Microorganisms are a fundamental component of the ecosystem. From the moment of birth, humans are in constant contact with them. Many of them engage in mutually beneficial relationships with humans; for example, the host supplies protection and nutrients, while they protect the host against pathogenic microorganisms [1]. This phenomenon is known as colonization resistance [2]. The human microbiota comprises the domains Eukarya, Bacteria, Archaea, and viruses. This microbiota can be found in all body niches. Therefore, humans are considered a holobiont. The holobiont refers to a complex biological unit consisting of a host and its diverse community of symbiotic microorganisms [3]. Symbiotic communities differ across body regions, and they can vary between individuals. Two of the main niches on which microbiota research has focused are the oral cavity and intestinal tract. Nevertheless, in recent years, the vaginal microbiota has received increased attention due to its involvement in vaginal health [4,5]. The vagina hosts a complex microecosystem; 16S rRNA sequencing has revealed that it contains approximately 10^10^ to 10^11^ microorganisms [6]. The microbiota is essential for maintaining vaginal health; unlike other microbiomes, the normal vaginal microbiome is relatively low in diversity, with the genus *Lactobacillus* predominating [4,7]. This host–microbiome interaction establishes a mutualistic relationship: the host provides nutrients, moisture, and a warm environment for the microbiome. In response, resident bacteria produce molecules that inhibit the growth of foreign bacteria and anti-inflammatory molecules [8]. The balance between the host and the microbiome can be modified by both internal and/or external factors. Internal factors include hormonal changes, age, and the functional state of the host’s innate and adaptive immune responses [8,9,10]. These internal changes alter the host environment, diminishing its ability to control opportunistic pathogenic bacteria, which may, in turn, lead to disease.

On the other hand, external factors include antibiotics, infections, and exposure to external microbiomes [11,12,13], all of which can alter the original microbiota. Variations in both internal and external factors may disrupt the host–microbiome ecosystem balance, leading to dysbiosis. In the case of the vagina, high diversity in the ecosystem has been correlated with bacterial vaginosis [7], which is associated with an increased risk of contracting a sexually transmitted infection (STI), elevated risk of pelvic inflammatory disease, increased risk of premature birth, and HPV infection, among others [14,15,16,17]. In the case of sexually transmitted infections, bacterial vaginosis increases the risk of acquiring herpes simplex virus type 2 (HSV-2), HPV, HIV, chlamydial infections, gonorrhea, and trichomoniasis [8]. In particular, for HIV, bacterial vaginosis is one of the main risk factors for infection with this virus [18]. It has been demonstrated that bacterial vaginosis is associated with an increased risk of pelvic inflammatory disease, whereas a normal microbiota shows no effect on the risk of developing this condition [19]. Bacterial vaginosis is one of the main risk factors for spontaneous abortion and is associated with maternal infectious morbidity and preterm birth [20]. The presence of bacterial vaginosis early in pregnancy is associated with preterm delivery and with infants of low birth weight [21]. Alterations in the normal vaginal microbiome are considered a risk factor for HPV infection. Several studies have reported an association between a non-*Lactobacillus*-dominated microbiome and HPV infection and persistence. Bacterial vaginosis has been associated with an increased risk of HPV acquisition and a reduced rate of HPV clearance [22,23,24].

In this review, we discuss the role of the vaginal microbiome, its variability, and its connection to health issues, particularly cervical cancer, including a focus on the Mexican population.

## 2. Vaginal Microbiome

### 2.1. Methods for Identifying the Vaginal Microbiome

The microbiome is essential for maintaining a healthy vagina, and unlike other microbiomes, the normal vaginal microbiome is characterized by low diversity and a predominance of Lactobacillus species [4,7]. In contrast, high diversity within the vaginal microbiome correlates with the presence of bacterial vaginosis [7]. Therefore, understanding the composition of the vaginal microbiome is of critical importance. To achieve this, various methods have been developed to identify the microorganisms that constitute the normal microbiome, as well as those associated with different disease states.

#### 2.1.1. Culture-Based Method

The traditional method for investigating the presence of bacteria or other microorganisms is culture. This process involves inoculating the sample onto specialized culture media, allowing for the identification and quantification of microorganisms [25]. Microbiome culture offers several advantages: microorganisms can be isolated from samples using appropriate media and culture conditions; in vitro propagation can be achieved; and biochemical characterization of different microorganisms can be performed [26], including the determination of morphological and metabolic characteristics, growth rates, and metabolite production, among other aspects [25]. Culture is necessary for performing antibiograms, which help determine the appropriate antibiotic to use in the event of an infection [27]. Culture also exhibits high specificity and sensitivity for detecting the presence and quantity of microorganisms and serves as the basis for assessing the identity of microorganisms present in the microbiome.

Despite being the traditional method for determining the identity of microorganisms present in a sample, culture has important limitations: not all microorganisms are cultivable [28], and it is difficult to detect organisms that require special culture conditions, such as anaerobiosis or specific nutrient requirements. Culture-based methods require long periods of time to obtain isolated microbial colonies [29]. All of these factors limit the study of such microorganisms.

Culturomics is a culture-based technique that relies on sophisticated culture methods, including a wide range of culture media and diverse culture conditions (varying temperature, atmosphere, salinity, pH, among others). The aim of these culture conditions is to promote the growth of the largest number of organisms. Once isolated, microorganisms are identified using MALDI–TOF mass spectrometry. Using this approach, the number of identified microorganisms has increased substantially; for example, in the human gut, 668 bacterial species and 2 archaeal species had previously been identified, whereas culturomics enabled the identification of 1057 prokaryotic species [30]. Despite the advantages of culturomics, this approach has certain limitations, such as increased time required to process samples and a greater workload, which restricts the number of samples that can be analyzed compared with other methods. As with traditional culture, culturomics cannot identify non-cultivable microorganisms and cannot provide information on gene expression or the functional roles of the identified organisms. Therefore, genomic sequencing of newly identified microorganisms remains necessary [30].

#### 2.1.2. 16S rRNA Sequencing

Metagenomics-based techniques (also referred to as culture-independent methods) are used to rapidly and accurately characterize microbiomes. These approaches require prior DNA isolation; once DNA is obtained, two main strategies are commonly employed. Shotgun metagenomic analysis focuses on sequencing all microbial DNA present in the sample. In contrast, marker gene sequencing is based on sequencing a specific genetic marker across all genomes in the sample. Among the most widely used markers is the 16S rRNA gene, which is specific to archaea and bacteria. In comparison, the 18S rRNA and 28S rRNA genes are specific to eukaryotic microorganisms, while the internal transcribed spacer (ITS) region is commonly used for fungi [31,32].

The 16S rRNA gene is one of the most conserved genes, making it an excellent target for studying bacterial diversity. It contains nine hypervariable regions that can be used to distinguish species based on individual nucleotide variation. This gene has been widely used in microbiology to identify bacteria and to delineate relationships at the species and strain levels [32,33]. The use of a single hypervariable region is not sufficient to differentiate all bacterial strains; therefore, multiple hypervariable regions must be analyzed to ensure accurate sample identification [32,34]. For the analysis of the vaginal microbiota, several hypervariable regions have been used, including V1–V2 [4], V1–V3 [5], and V3–V4 [35]. The best results have been obtained using concatenated analyses of the V2, V3, V4, and V6–V7 regions [36].

The main advantages of using 16S rRNA gene sequencing are as follows: it does not require culture methods, enabling the study of non-culturable microorganisms, providing a broad profile of the sample [37]; high throughput with a favorable cost–benefit ratio, next-generation 16S sequencing allows for simultaneous analysis of many samples and multiple taxa at a relatively low cost [38]. Since 16S sequencing has been widely used for years, a curated reference database exists for taxonomic classification of samples [37]. Despite being a widely used technique, it still presents limitations that must be taken into account: short fragment sequencing typically cannot discriminate between very closely related species, because taxonomic resolution stops at the genus level [38,39]. PCR amplification can bias community composition since primers bind differently across taxa and certain variable regions are preferentially amplified [40]. This sequencing provides taxonomic profiles only and does not reveal functional gene content or metabolic potential [37,38]. Technical and analytical analyses can be complex; data analysis, clustering choice, and bioinformatics pipelines all influence outcomes and comparability across studies [41].

The method used to determine microbiome composition can affect the accuracy of the results; therefore, it is essential to understand the advantages and limitations of the available techniques. Culturomics combines culture with mass spectrometry detection, and this approach can increase the number of identified organisms; however, it is a time-consuming process and restricts identification to cultivable microorganisms. In contrast, 16S rRNA gene sequencing does not require culture, is relatively fast to perform, allows for the analysis of large numbers of samples, and benefits from curated databases for data comparison. Nevertheless, its limitations may introduce bias and affect reproducibility. The selection of the most appropriate method will depend on each laboratory, taking into account cost and technical capacity.

### 2.2. Geographical and Individual Variation

Defining what constitutes a normal vaginal microbiome can be problematic because women from different countries exhibit distinct microbiome features, with some variations between regions; for example, for healthy women in Canada, the main species are *Lactobacillus* (*L.*) *crispatus*, *L. inners* and *L. jensenii* [41]; the microbiome of healthy Chinese women is dominated by *Lactobacillus gasseri*, *L. iners*, and *L. crispatus*, whereas in the Indian population, the dominant species were *L. crispatus*, *L. gasseri*, and *L. jensenii* [42]; in contrast, in Mexican women, *L. acidophilus*, *L. iners*, *L. gasseri* and *L. delbrueckii* predominate [43]. The microbiome is not static; it changes over time. In particular, the vaginal microbiome experiences fluctuations throughout the menstrual cycle and across a woman’s lifespan. For example, differences in microbiome composition have been observed between pregnant and non-pregnant women. In pregnant women, *Lactobacillus* spp., *Actinomycetales*, *Clostridiales*, and *Bacteroidales* predominate. In contrast, in non-pregnant women, the microbiome is dominated by *Lactobacillus* spp., *Actinobacteria*, *Prevotella*, *Veillonellaceae*, *Streptococcus*, Proteobacteria, Bifidobacteriaceae, Bacteroides, and Burkholderiales [44]. The vaginal morphology (stratified non-keratinized squamous epithelium covered by cervicovaginal secretions) [8] and physiology (oxygen and nutrients are derived from submucosal tissues) [45] result in the vaginal environment being relatively anaerobic; these conditions create a favorable environment for the microbiome, promoting host–microbe symbiosis and resulting in the establishment of what is referred to as the vaginal microbiome [8,46].

### 2.3. Community State Type (CST) Classification

For clarity, the Human Microbiome Project has established a reference microbiome based on what is considered a relatively healthy adult, and a healthy microbiome is defined as one present in the absence of evident disease [12,47]. Studies have been conducted to determine the normal composition of the vaginal microbiome. Samples were obtained from 396 relatively healthy women from the USA of different ethnicities, and 16S rRNA gene sequencing was performed. Based on species abundance, the vaginal microbiome can be classified into five community state types (CST) [4,48]. In most of these communities, *Lactobacillus* species are dominant: in CST-I, *L. crispatus* predominates; in CST-II, *L. gasseri* predominates; *L. iners* predominates in CST-III; and *L. jensenii* predominates in CST-V [4]. In contrast, CST-IV is characterized by a lower abundance of *Lactobacilli* and a higher proportion of anaerobic bacteria under pathological conditions [49]. Subsequently, CST-IV was further subdivided into CST-IV A (*Anaerococcus*, *Peptoniphilus*, *Corynebacterium*, *Prevotella*, *Finegoldia*, and *Streptococcus*), CST-IV B (*Atopobium*, *Gardnerella*, *Sneathia*, *Mobiluncus*, *Megasphaera*, and *Clostridiales*) [50], and CST-IV C, which, in turn, has been subdivided into five subgroups: CST-IV C0 (moderate proportion of *Prevotella*), CST-IV C1 (dominated by *Streptococcus*), CST-IV C2 (*Enterococcus*), CST-IV C3 (*Bifidobacterium*), and CST-IV C4 (*Staphylococcus)* [51] (Table 1).

### 2.4. Lactobacillus-Mediated Protective Mechanisms

Of the total human microbiota, the urogenital region (which includes the vagina) accounts for approximately 9% of the overall composition [52]. In a healthy microbiome, the principal component is typically one of four *Lactobacillus* species: *L. crispatus*, *L. jensenii*, *L. gasseri*, and *L. iners* [53,54]. These bacteria are capable of protecting the vagina from foreign bacteria and other pathogens through different mechanisms: competition for nutrients and competition for adherence to the tissue, decreased vaginal pH, production of bacteriocins, secretion of lactic acid, and modulation of the immune system, among others [55]. One of the main characteristics of *Lactobacilli* is the production of lactic acid; these bacteria produce both L- and D-lactic acid and are the primary source of these molecules [56,57]. In contrast, epithelial cells produce approximately 20% of the total L-lactic acid [58]. Of the two lactic acid isomers, the D-isomer plays a more relevant role in protection than the L-isomer [56,59,60]. A scarcity or absence of D-lactic acid results in increased levels of EMMPRIN (Extracellular Matrix Metalloproteinase Inducer), leading to high concentrations of MMP-8 (matrix metalloproteinase-8). This facilitates extracellular matrix degradation and allows for bacterial migration toward the endocervix and into the uterus [56].

**Figure 1:**
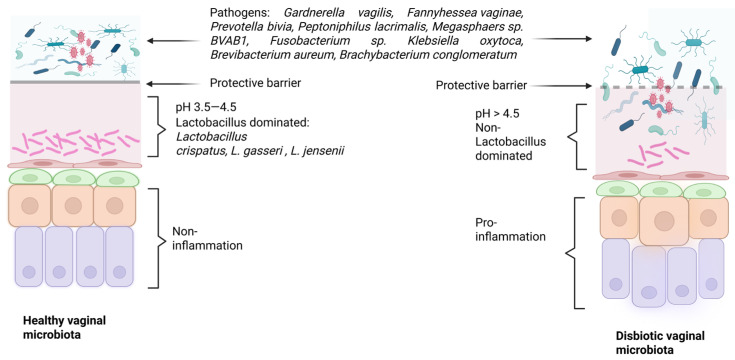
Healthy and dysbiotic vaginal microbiota. The normal vaginal microbiome is dominated by *Lactobacillus* species (*L. crispatus*, *L. gasseri*, *L. jennseni*, among others). These bacteria maintain a low pH and protect against the invasion of pathogenic microorganisms. When the vaginal microenvironment undergoes dysbiosis, *Lactobacillus* species cease to dominate, the pH becomes alkalinized, and pathogenic microorganisms (such as *Gardnerella vaginalis*, *Fannyhessea vaginae*, *Prevotella bivia*, *Peptoniphilus lacrimalis*, and *Megasphaera* spp., among others) can colonize the vagina.

All lactic acid produced acidifies the vaginal milieu, thereby inhibiting the growth of potential pathogens and promoting the proliferation of *Lactobacilli* themselves [61,62]. This secreted lactic acid acidifies the vaginal milieu (usually maintaining a pH between 3.5 and 4.5), which can protect against some viruses (e.g., HPV, HIV), certain bacteria (e.g., *N. gonorrhoeae*), and parasites (*T. vaginalis*) [63,64,65,66]. However, lactic-acid-mediated protection involves more than just acidification of the milieu: it also exhibits anti-inflammatory properties in vitro, and the presence of an optimal microbiome dominated by *Lactobacillus* is associated with a low-inflammatory environment (in vivo and in vitro) [67,68,69,70]. Not all *Lactobacillus* species behave the same way: *L. iners* has been associated with dysbiosis and low microbiota stability, and it cannot produce D-lactic acid. In contrast, *L. crispatus* is associated with a healthy and stable vaginal microbiome and is capable of producing both D- and L-lactic acid [71]. Another way in which *Lactobacilli* protect against potential pathogens is through the production of bacteriocins, antimicrobial peptides that can permeabilize the membranes of their targets, which include certain *Gardnerella* species [72,73] (Figure 1).

*Lactobacilli* can form a biofilm, a protective barrier that adheres to the epithelium, which inhibits pathogen adhesion. Complementing this defense, they also produce biosurfactants and hydrogen peroxide, which act as antimicrobial agents [74,75]. Although *Lactobacilli* have been reported to produce H_2_O_2_, their role in vaginal protection continues to be controversial: some studies show that H_2_O_2_ inhibits the growth of pathogenic bacteria [76,77]. Other studies suggest that H_2_O_2_ produced by *Lactobacilli* is not a protective mechanism because hypoxic conditions in the vagina hinder its production; it is possible that the high antioxidant capacity of vaginal fluid impedes the bactericidal activity of H_2_O_2_. H_2_O_2_ is more likely toxic to *Lactobacilli* than to pathogenic bacteria [78,79,80]. Another form of protection mediated by *L. crispatus* involves its regulation of specific biochemical pathways, such as the biosynthesis pathways of L-lysine, L-threonine, and L-methionine, which have been associated with cancer prevention [81]. L-lysine has been associated with anti-inflammatory activity and reduced oxidative stress [81,82]. Under inflammatory conditions, threonine can regulate immune cell differentiation, cytokine expression, and signaling cascades related to the immune response (MAPK, mTOR), including the target of rapamycin (TOR), thereby contributing to the maintenance of tissue health [83,84]. L-methionine exerts both direct and indirect antioxidant functions [85], and several studies have also shown that methionine has anti-inflammatory effects [86,87].

On the other hand, in women with dysplasia, *G. vaginalis* and *Fannyhessea vaginae* contribute to the peptidoglycan and L-alanine biosynthesis pathways, both of which are associated with cervical cancer [81].

## 3. HPV and Microbiome

### 3.1. HPV Mechanism and Host Immunity

**Figure 2:**
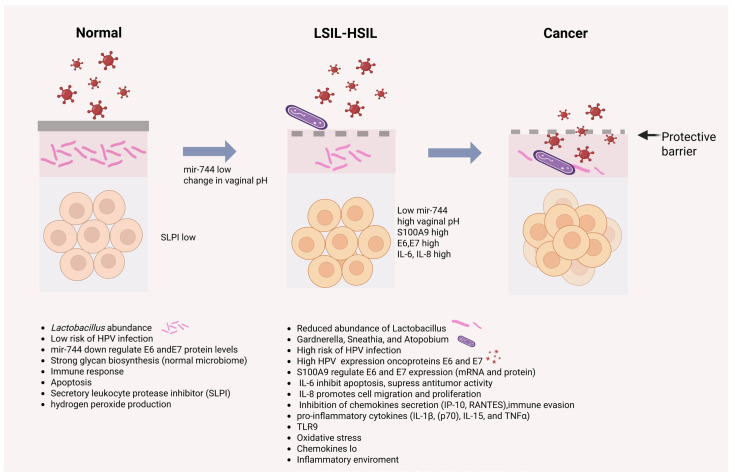
HPV and microbiome. The presence of a normal microbiome, particularly the dominance of *Lactobacillus*, protects against HPV infection through multiple mechanisms: high concentrations of lactic acid (especially D-lactic acid) induce the expression of miR-744, which inhibits the expression of the E6 and E7 oncoproteins; glycan synthesis by the normal microbiome provides additional protection against HPV infection. Other protective mechanisms include immune response activation, apoptosis, SLPI, and H_2_O_2_. A reduction in *Lactobacillus* abundance and the presence of anaerobic bacteria alter the microenvironment, making it more conducive to HPV infection. Decreased lactic acid concentrations reduce miR-744 expression and result in an elevated vaginal pH, thereby promoting a proinflammatory environment characterized by cytokines such as IL-6, which can inhibit apoptosis and suppress antitumor activity, and IL-8, which promotes cell migration and proliferation. In addition, chemokine secretion is inhibited, facilitating immune evasion; the cytokine S100A9 is expressed, inducing the expression of the E6 and E7 oncoproteins; and oxidative stress is increased. Altogether, these changes promote the malignant transformation of cells. LSIL (low-grade squamous intraepithelial lesion; HSIL (high-grade squamous intraepithelial lesion).

Human papillomavirus (HPV) is an infectious agent that is primarily transmitted through sexual contact and can cause HPV-associated cancers in both men and women. Nearly all cervical cancers are associated with HPV [88]. HPVs are classified into low- and high-risk types; low-risk types can cause benign lesions, whereas high-risk types promote premalignant and malignant changes. Specifically, HPV-16 and HPV-18 are high-risk oncogenic types, responsible for approximately 60% and 15% of cervical cancer cases, respectively [89,90]. The HPV viral oncoproteins E6 and E7 lead to the degradation of p53 and pRb, respectively, resulting in entry into the S phase without G1 arrest [88]. These high-risk HPV viral oncogenes disrupt numerous cellular processes, including DNA repair, angiogenesis, and/or apoptosis, ultimately leading to carcinogenesis [88]. Both the innate and adaptive immune systems protect against pathogens that target mucosal surfaces, such as HPV [91]. An association has been observed between HPV infection and cytokine responses, implicating the cellular immune response in the control and/or elimination of the infection. Some of the cytokines involved include MCP-1 and IL-8, among others [92,93]. The cytokine-mediated response occurs within days after the initial HPV infection and normally results in clearance of the infection [94,95,96]. Among the protective effects mediated by *Lactobacillus*, this bacterium can inhibit the expression of the viral oncoproteins E6/E7, thereby reducing proliferation and inducing apoptosis of the infected cell. A *Lactobacillus*-dominated microbiome enhances protection, strengthens the local immune response, and reduces the risk of HPV infection (Figure 2) [97,98,99].

### 3.2. Microbiome Shifts During CIN Progression

Among all *Lactobacillus* species, *L. crispatus* is considered the most beneficial. A high abundance of this bacterium is associated with a lower presence of high-risk HPV [100,101], whereas a decrease in *L. crispatus* abundance is linked to an increase in cervical lesions [102]; these reduced levels of *L. crispatus* are also found in patients with high-risk HPV infection, low-grade squamous intraepithelial lesion (LSIL), high-grade squamous intraepithelial lesion (HSIL), and cervical carcinoma [100,103,104,105,106]. In LSIL (CIN1), several bacteria have been identified, predominantly *G. vaginalis.* Additionally, *Prevotella bivia*, *L. iners*, *Peptoniphilus lacrimalis*, *Megasphaera* sp., and, to a lesser extent, the genus *Sneathia* can also be found [105,107,108,109]. In HSIL (CIN2, 3), the presence of *L. iners* [105,108], *G. vaginalis Ercella*, *Bacillus*, *B. lautia* and *Terrisporobacter* has been reported [110], along with a high prevalence of *Megasphaera* [107,110]. To a lesser extent, *Alloscardovia omnicolens*, *Staphylococcus aureus*, and *Candidatus endolissoclinum* have also been identified [81,111,112].

When dysbiosis occurs in the vaginal microbiome, certain bacteria can promote HPV progression by inducing inflammation, which is one of the conditions required for HPV infection, increasing the likelihood of developing cervical intraepithelial neoplasia (CIN) and the development of invasive cancer. A reduction in *Lactobacillus* abundance is also associated with CIN development, fostering a pro-inflammatory environment that enhances the expression of HPV oncoproteins E6 and E7 [113,114]. In addition to the role of the inflammatory environment, miR-744 (a lactic-acid-inducible miRNA) [115] and the proinflammatory cytokine S100A9 [114] regulate the expression of E6 and E7 (Figure 2).

Among the pro-inflammatory cytokines associated with dysbiosis and bacterial vaginosis are IL-6, IL-8, and TNF-α, among others. This enhances oxidative stress, increasing the risk of progression towards high-grade CIN. Consequently, elevated levels of these pro-inflammatory cytokines are commonly observed in women with dysbiosis [99,113]. Some studies have shown that women with a low abundance of *Lactobacillus* and a high abundance of *Gardnerella*, *Sneathia*, and *Atopobium* are less likely to clear HPV infection [116,117,118,119]. In contrast to *Lactobacillus*, anaerobic bacteria associated with dysbiosis can diminish the innate immune response and inhibit apoptosis, thereby facilitating HPV infection. Anaerobic bacteria stimulate the proinflammatory innate immune response, promoting the secretion of cytokines such as IL-6 and IL-8. These cytokines mediate diverse responses; for example, IL-6 inhibits apoptosis [120] and suppresses antitumor immunity [121], whereas IL-8 promotes cell migration and proliferation [122]. Another consequence of the presence of anaerobic bacteria is the inhibition of chemokine secretion, such as IP-10 or RANTES, which results in impaired chemotaxis and may contribute to immune evasion [123]. As a result, the production of secretory leukocyte protease inhibitor (SLPI), which protects the mucosa against infections, is reduced [97,113]. Bacteria associated with dysbiosis can also increase vaginal pH and reduce hydrogen peroxide production, which may lead to mucosal damage and consequently increase the risk of HPV entry [116,118]. This suggests that while, as stated in Section 2, the role of H_2_O_2_ in protection against pathogenic bacteria is controversial and high levels of H_2_O_2_ may be toxic to Lactobacilli, H_2_O_2_ levels need to be maintained at biologically appropriate levels to maintain healthy vaginal tissue. Therefore, vaginal dysbiosis leads to a decrease in the immune response of the cervix while promoting the colonization of foreign microorganisms [124], increasing the presence of pro-inflammatory cytokines (IL-1β, IL-12 (p70), IL-15, and TNFα) and regulatory cytokines (IL-2 (p40) [125]. One effect of dysbiosis is the increased expression of TLR9, which coincides with the progression of cervical lesions, particularly in women positive for HPV16 [112]. Yang et al. propose that strong glycan biosynthesis in the normal microbiome may be one mechanism that helps to resist dysbiosis and HPV infection [126].

### 3.3. The Use of Probiotics (Lactobacillus) as Treatment

The treatment of precancerous lesions, particularly HSIL, can influence the composition of the microbiota. The most commonly used therapy for HSIL is a loop electrosurgical excisional procedure (LEEP) [127]. This treatment induces the re-establishment of *Lactobacilli* dominance in the vaginal microbiome. A transition from *Prevotella* spp. and *Sneathia* spp. (CST-IV) to *L. iners* (CST-III) was observed three months after treatment [128]. Another study showed that *L. crispatus* (CST-I) increased, while CST-IV bacteria decreased six months after LEEP in patients who cleared HPV. In contrast, patients with persistent HPV infection did not exhibit any changes [129]. *Lactobacilli* can be used as a treatment (probiotic), as they are capable of promoting a healthy vaginal microbiome.

#### 3.3.1. Oral Administration

Oral administration of *L. crispatus* M247 in HPV-positive patients (CST-IV) resulted, after 90 days of treatment, in a 70% reduction in HPV presence [130]. In addition, a significant shift in CST composition was observed, with 94% of patients being classified as CST-I [130]. Dellino et al. administered *L. crispatus* M247 (20 × 10^9^ colony-forming units, CFUs) orally for approximately 12 months. HPV-positive patients showed a higher rate of lesion regression and HPV clearance compared to the control group [131]. Other *Lactobacillus* strains administered orally include *L. casei* Shirota and a combination of *L. rhamnosus* GR-1 (50%) with *L. reuteri* RC-14 (50%). The dose of *L. casei* Shirota was 8 × 10^9^ CFU/day for six months, with improved HPV clearance after six months of treatment compared with the control group [132]. For the combination of *L. rhamnosus* GR-1 (50%) and *L. reuteri* RC-14 (50%), the dose used was 5.4 billion CFUs per tablet, and HPV presence was assessed at 3, 6, 9, and 12 months. No significant differences were found in viral presence between the experimental groups and the control group. However, an improvement was observed in cervical smears, with a reduction in the proportion of slightly abnormal or unsatisfactory cytological smears [133].

#### 3.3.2. Vaginal Administration

Similar results have been observed when using the intravaginal route to administer the strain *L. crispatus* chen-01; this treatment reduced HPV presence, enhanced HPV clearance, and improved vaginal inflammation [134]. The combination of probiotics and antibiotics is a promising strategy to reduce the risk of CIN. Treatment with metronidazole or clindamycin can reduce the diversity of vaginal microbiomes; however, it is necessary to repopulate the microbiome with beneficial bacteria. To achieve this, the probiotic LACTIN-V, which contains the *L. crispatus* CTV-05 strain, has been used. This treatment reduces the recurrence of bacterial vaginosis after metronidazole therapy [135,136]. Another strain that has been used is *L. rhamnosus* BMX54 (100,000 CFU) administered for three and six months, with outcomes evaluated at nine months. Both treatment regimens showed improvements in cytology and viral clearance [137]. The combined use of *Lactobacillus* and interferon alpha-2B (gel) for three months resulted in a significantly higher HPV clearance compared with patients who received interferon alone [138].

## 4. Microbiome in the Mexican Population

In Mexico, several studies have been conducted on the vaginal microbiome. In 2011, Hernández-Rodríguez et al. identified the microbial communities of healthy pregnant Mexican women in Mexico City, finding that the genus *Lactobacillus* was the most represented, being present in 98% of the samples. *L. acidophilus* predominated in 78% of the samples, followed by *L. iners* (54%), *L. gasseri* (20%), and *L. delbrueckii* (6%). They also identified 17 microorganisms associated with bacterial vaginosis, among which *Ureaplasma urealyticum* was the most represented (21%), followed by BVAB1 (*Bacterial Vaginosis-Associated Bacterium* 1, or [*Candidatus*] *Lachnocurva vaginae*) (17%) [43]. Samples in this study were collected at different times during pregnancy. González-Sánchez et al. were more specific, obtaining samples during the third trimester of pregnancy and subsequently at active term labor in patients from Mexico City and the metropolitan area. The genus *Lactobacillus* dominated most samples (80%) at both times, while the remaining 20% showed high abundances of *Gardnerella*, *Prevotella*, and *Atopobiaceae.* However, no statistically significant differences were observed between the third trimester and active term labor. Nonetheless, a trend was noted at active term labor, characterized by higher absolute counts of *Gardnerella*, *Faecalibaculum*, *Ileibacterium*, and *Lactococcus*, and lower absolute counts of the genus *Lactobacillus* [139]. In 2013, Martínez-Peña et al. identified *Lactobacillus* strains present in vaginal secretions from healthy non-pregnant Mexican women in Mexico City. The *Lactobacillus* species identified were *L. gasseri*, *L. fermentum*, *L. rhamnosus*, *L. jensenii*, *L. crispatus*, and *L. brevis* [140].

As mentioned above, dysbiosis can induce alterations in cytokine expression. Audirac-Chalifour et al. assessed cytokine profiles and correlated them with the vaginal microbiome and the clinical stage of cervical cancer. The samples were obtained from the state of Morelos and Mexico City. They found a significant difference in microbial diversity between HPV-negative women and those with SIL (squamous intraepithelial lesion) or cervical cancer, with the latter showing the most significant variation. Regarding dominant bacterial taxa, *Lactobacillus crispatus* and *L. iners* were prevalent in healthy women, whereas *Sneathia* spp. predominated in SIL, and *Fusobacterium* in cervical cancer. In the latter case, high levels of IL-4 and TGF-β1 mRNA were also detected [141].

Manzanares-Leal et al. identified aerobic microbiota in women with cervical cancer from Mexico City. Two study groups were included: one with advanced cervical cancer and another without cervical cancer. In both groups, facultative aerobic and enteric bacteria were detected, primarily *Staphylococcus epidermidis*, *Streptococcus agalactiae*, *Enterococcus faecalis*, *Escherichia fergusonii*, and *Corynebacterium amycolatum.* Seven species were identified as specific to the group of women with cervical cancer: *Streptococcus urinalis*, *Escherichia coli*, *Bacillus safensis*, *Bacillus malikii*, *Corynebacterium jeikeium*, *Corynebacterium striatum*, and *Lactobacillus rhamnosus.* In contrast, eight species were detected exclusively in the group of women without cervical cancer, which were absent in the cancer group: *Staphylococcus pasteuri*, *Staphylococcus auricularis*, *Staphylococcus capitis* subsp. *capitis*, *Facklamia hominis*, *Paenibacillus urinalis*, *Pseudocitrobacter faecalis*, *Brevibacterium masiliense*, and *Klebsiella oxytoca* [142]. Although this is a preliminary study, it reveals differences in the aerobic bacterial profiles, with some species being exclusive to cervical cancer cases and others detected only in women without cancer. Further research is required to elucidate the behavior of these bacteria and their potential role in the development of cervical cancer.

Mulato-Briones et al. employed a culturomics approach—which combines culture-based techniques with Vitek mass spectrometry and 16S rDNA sequencing—to investigate differences in the microbiota between women without cervical cancer and those with cervical cancer in patients recruited in Mexico City. They found a clear difference in microbiota composition between patients without cancer and those with cervical cancer. Four microbial community groups were isolated: (i) *Lactobacillus* only, (ii) *Lactobacillus* plus *Staphylococcus*, (iii) *Staphylococcus* plus *Streptococcus*, and (iv) a group dominated primarily by Proteobacteria. In addition, *Enterococcus faecalis* and *Escherichia coli* were observed. In the cervical cancer group, the phyla Firmicutes and Proteobacteria predominated, with a notable absence of *Lactobacillus* and an increased diversity of anaerobic and opportunistic microbiota. The most frequently detected genera were *Streptococcus* and *Staphylococcus*, followed by *Enterococcus*, *Paenibacillus*, and *Gemella.* A wide variety of Proteobacteria was also identified, including *E. coli*, *Acinetobacter*, *Campylobacter*, and *Citrobacter.* Regarding clinical stage, early-stage cancer was associated mainly with *Corynebacterium*, *Streptococcus*, *Escherichia*, and *Staphylococcus*, with an absence of strict anaerobes. In contrast, advanced stages were characterized primarily by strict anaerobic bacteria [143]. These findings indicate that the increased diversity of the microbiota is associated with the presence of cervical cancer and that strict anaerobic bacteria are correlated with advanced clinical stages. The studies cited above also support the premise mentioned earlier that probiotic treatments, including *Lactobacillus*, may serve as an adjuvant to improve patient outcomes.

Nieves-Ramírez et al. demonstrated that the presence of *Brevibacterium aureum* and *Brachybacterium conglomeratum* is associated with HPV16 infection and/or SIL [144]. Subsequently, in 2023, Cortés-Ortiz et al. identified that *Brachybacterium conglomeratum*— previously regarded primarily as an environmental bacterium—is associated with HPV and cervical infections in Mexican patients. Samples were obtained from women in Ixtapaluca, State of Mexico, and categorized into three groups: patients with LSIL, patients with cervicovaginal infection, and patients with precancerous lesions. *B. conglomeratum* was detected across all groups, with the highest prevalence in LSIL cases. This group also exhibited coinfection with multiple high-risk HPV types. Notably, *B. conglomeratum* was not found as a solitary pathogen but rather in association with *Gardnerella vaginalis*, *Atopobium vaginae*, and *Ureaplasma parvum* [145]. These results highlight the complex interactions between the microbiota, dysbiosis, and precancerous lesions. *B. conglomeratum* is not found in LSIL as a solitary organism; rather, its presence appears to require the coexistence of other pathogens, which may ‘prime’ or alter the local environment, thereby enabling colonization by bacteria traditionally regarded as environmental species.

Sánchez-García et al. evaluated the association between normal microbiota and pathogenic organisms in women with asymptomatic vaginal dysbiosis in Tabasco, Mexico. Women with bacterial vaginosis (BV) showed an increased prevalence of *Chlamydia trachomatis* and *Mycoplasma hominis.* Regarding BV-associated organisms, *Gardnerella vaginalis* was linked to *C. trachomatis* and/or *Ureaplasma parvum.* In contrast, *Atopobium vaginae* and *Megasphaera* type 1 correlated with *M. hominis*. This study also reported a slightly higher, though not statistically significant, prevalence of HPV in women with BV [146]. In contrast, Romero-Morelos et al. analyzed samples obtained from Taxco, Guerrero. They proposed that *Gardnerella vaginalis* and *Atopobium vaginae* may represent potential components of the normal microbiota in the studied population, as both species were detected in healthy women and in patients with precursor lesions. Furthermore, no association was found between the presence of these bacteria and HPV infection [147] (Table 2).

Importantly, these findings do not contradict the description of healthy and dysbiotic vaginal microbiota shown in Figure 1. In the healthy vagina, pathogenic microbes are present in the healthy microbiome, but are not in direct contact with vaginal tissue. Importantly, it is possible that some of these pathogenic microbes have a role in maintaining a normal healthy microbiota. Consequently, the interaction of *G. vaginalis* and *A. vaginae*, and other possible pathogenic microbes, with the overlying mucosal barrier and with the underlying tissue need to be assessed in future studies.

Collectively, these studies indicate that while CST-II and CST-III are common in Mexican women without overt disease, progression to SIL and cervical cancer is associated with a transition toward CST-IV. This distribution contrasts with global cohorts, where CST-I predominates in health, underscoring the need to interpret CST-based risk models within a population-specific framework and reinforcing the public health relevance of defining CST distributions tailored to Mexican women.

It is important to acknowledge that studies conducted in Mexico encompass heterogeneous populations, including pregnant women, asymptomatic individuals, and patients with precancerous lesions or advanced cervical cancer. These clinical and physiological conditions are known to profoundly influence vaginal microbiome composition, as hormonal status, immune modulation during pregnancy, cancer-associated inflammation, treatment exposure, ethnicity, and socioeconomic factors all shape microbial community structure.

One of the main challenges is the centralization of studies: the vast majority have been conducted in Mexico City and its metropolitan area, with very few in other regions. Given the substantial population diversity in Mexico, restricting research to the capital excludes most of the population. It is therefore necessary to develop a national project to characterize both the normal microbiome and disease-associated microbiomes to understand their potential interactions and, if appropriate, to develop probiotics tailored to the specific characteristics of the Mexican population to complement conventional treatments.

## 5. Conclusions

Cervical cancer remains a public health problem affecting women, especially in developing countries. Persistent high-risk HPV infection is the main trigger of the development of cervical cancer. Recently, the focus has turned to the role of the vaginal microbiome. The vaginal microbiome plays a fundamental role in maintaining a healthy environment, preventing infections caused by pathogenic microorganisms and viruses such as HIV and HPV. Regarding the latter, the microbiome, and, in particular, *Lactobacillus* species display mechanisms that promote an anti-inflammatory milieu, thereby inhibiting the expression of viral oncoproteins E6/E7 in infected cells. Thus, it may be helpful to complement conventional treatments with probiotics containing *L. crispatus*. More research is needed about the interactions between the microbiome and HPV in the cervix that lead to cervical cancer. One approach could be analyzing the responses of cervical cancer culture-based techniques to different species of *Lactobacillus* to gain valuable insights to improve therapeutic approaches and reverse dysbiosis, ultimately leading to better outcomes for women suffering from these conditions and perhaps reducing the risk of cancer development.

## Figures and Tables

**Table 1:** Community state types of vaginal microbiome.

Community State Types (CST) *	Dominant Species	Associated Health Status	Associated Pathologies
CST-I	*Lactobacillus crispatus*	Highly healthy and stable	Lower risk of infections (HPV) and preterm birth
CST-II	*Lactobacillus gasseri*	Healthy and stable	Lower risk of sexually transmitted infections (STI) and preterm birth
CST-III	*Lactobacillus iners*	Healthy, less stable	Greater tendency to dysbiosis; coexists with pathogens
CST-V	*Lactobacillus jensenii*	Healthy	Absence of pathologies
CST-IV	Lower abundance of *Lactobacillus*; high proportion of anaerobic bacteria	Dysbiosis	Bacterial vaginosis, STIs, candidiasis, viral infections (HPV, HIV)
CST-IV	CST-IV A *Anaerococcus*, *Peptoniphilus*, *Corynebacterium*, *Prevotela*, *Finegoldia*, *Streptococcus* CST-IV B *Atopobium*, *Gardnerella*, *Sneathia*, *Mobiluncus*, *Megasphaera*, *Clostridiales* CST-IV C Varies by subgroup Divided into five subgroups	Dysbiosis	Bacterial vaginosis
	CST-IV C0 *Prevotella* CST-IV C1 *Streptococcus* CST-IV C2 *Enterococcus* CST-IV C3 *Bifidobacterium* CST-IV C4 *Staphylococcus*	Dysbiosis	Bacterial vaginosis

* This table summarizes the classification of the vaginal microbiome into different community state types. The data is based on studies that sequenced the 16S rRNA gene in samples from women to determine the dominant bacterial species.

**Table 2:** Bacterial species identified in the Mexican population.

Author Region	Identified Strain * Healthy	Identified Strain * Disease	Sample Characteristic
Hernández-Rodríguez et al. [43] Mexico City	*L. acidophilus* (predominant) *L. iners*, *L gasseri* *L. delbrueckii*	Bacterial vaginosis: *Ureaplasma urealyticum*	Samples collected during pregnancy
		BVAB1 (Bacterial vaginosis associated bacteria	
González-Sánchez et al. [139] Mexico City (Metropolitan area)	*Lactobacillus* (predominant) *Gardnerella*, *Prevotella* Atopobiaceae	No data	Samples collected at the third trimester of pregnancy
Martínez-Peña et al. [140] Mexico City	*L. gasseri*, *L. fermentum*, *L. rhamnosus*, *L. jensenii*, *L. crispatus* (low frequency) *L. brevi*	No data	Samples from healthy non-pregnant women
Audirac-Chalifour et al. [141] Mexico City and State of Morelos	*L. crispatus* *L. iners*	*Sneathia* spp. (predominant in SIL) *Fusobacterium* (in cervical cancer)	SIL and women with normal colposcopy (State of Morelos)
			Cervical carcinoma (Mexico City)
Manzanares-Leal et al. [142] Mexico City	*Staphilococcus pasteuri*, *Staphilococcus auricularis*, *Staphilococcus capitis* subsp. *capitis*, *Facklamia hominis*, *Paenibacillus orinalis*, *Pseudocitrobacter faecalis*, *Brevibacterium masiliense*, *Klebsiella oxytoca*	Cervical cancer: *Streptococcus urinalis*, *Escherichia coli*, *Bacillus safensis*, *Bacillus maliki*, *Corynebacterium jeikeium*, *Corynebacterium striatum*, *L. rhamnosus*	Identified aerobic microbiome in women with and without cervical cancer
Mulato-Briones et al. [143] Mexico City	(i) *Lacobacillus* only, (ii) *Lactobacillus* plus *Staphylo coccus* (iii) *Staphylococcus* plus *Streptococcus* (iv) A group dominated primarily by Proteobacteria Most representative: *L. jensenii*, *L. crispatus*	*Streptococcus*, *Staphylococcus*, *Enterococcus*, *Paenibacillus*, *Gemella* Proteobacteria: *E. coli*, *Acinetobacter*, *Campylobacter*, *Citrobacter* Early-stage cancer: *Corynebacterium*, *Streptococcus*, *Escherichia*, *Staphylococcus*, with an absence of strict anaerobes	50 non-cancer women 49 women with cervical cancer
Nieves-Ramírez et al. [144] Mexico City	No data	*Brevibacterium aureum*, *Brachybacterium conglomeratum* Associated with HPV16 infection and/or SIL	Samples from LSIL and HSIL
Cortés-Ortiz et al. [145] State of Mexico	No data	*Brachybacterium* *conglomeratum* (cervicovaginal lesion, LSIL, precancerous lesion) *Gardnerella vaginalis*, *Atopobium vaginae*, *Ureaplasma parvum*	Cervicovaginal lesions, LSIL, precancerous lesions
Sánchez-García et al. [146] Tabasco	No data	Bacterial vaginosis: Increased prevalence of *Chlamydia trachomatis* and *Mycoplasma hominis*	Women recruited during their routine gynecological inspection
Romero-Morales et al. [147] Guerrero	*Gardnerella vaginalis* *Atopobium vaginae*	*Gardnerella vaginalis* *Atopobium vaginae*	Samples without colposcopy and cytological alterations Precancerous lesions

* This table summarizes the bacterial species identified in the healthy population and in cervical cancer patients in some regions of Mexico.

## Data Availability

No new data were created or analyzed in this study. Data sharing is not applicable to this article.
